# Benefits and concerns associated with blockchain-based health information exchange (HIE): a qualitative study from physicians' perspectives

**DOI:** 10.1186/s12911-022-01815-8

**Published:** 2022-03-28

**Authors:** Pouyan Esmaeilzadeh

**Affiliations:** Department of Information Systems and Business Analytics, College of Business, Florida International University (FIU), Modesto A. Maidique Campus, 11200 S.W. 8th St, RB 261B, Miami, FL 33199 USA

**Keywords:** Blockchain technology, HIE, Physicians, Barriers, Benefits, Interviews

## Abstract

**Background:**

Blockchain technology has the potential to revolutionize information sharing in companies. Many studies suggest using blockchain-powered platforms to replace existing mechanisms for health information exchange (HIE) across healthcare organizations. However, very few blockchain-based projects have been implemented in the healthcare sector. This study takes a qualitative approach to explore benefits, concerns, and barriers to the rollout of blockchain in HIE projects from physicians' perspectives.

**Methods:**

The Promoting Action on Research Implementation in Health Services (PARIHS) framework was used to help us better understand root causes, existing problems, perceived risks, perceived benefits, and suggestions. In-depth interviews have been conducted with 38 physicians in six months. The data were analyzed and coded using NVIVO to classify conceptually similar themes mentioned by the interviewees.

**Results:**

In total, seven themes have been identified. The key benefits are categorized into three themes: innovative technological features, collaborative ecosystem, and system performance. The main concerns and risks are categorized into four themes: individual, organizational, technological, and market-related issues. The findings can contribute to knowledge by highlighting key values expected from blockchain technology in HIEs. The results also explore obstacles to leveraging the blockchain in healthcare from the perspectives of an important stakeholder (physicians).

**Conclusions:**

The results show that although blockchain technology may create several benefits (e.g., innovative technological features, collaborative ecosystem, and system performance), its applications in healthcare are still in their early stages. The perceptions of the individual issues (e.g., lack of knowledge), organizational issues (e.g., implementation issues), technological issues (e.g., blockchain model types), and market-related issues (e.g., regulatory concerns) indicate that blockchain-based applications in healthcare continue to be an emerging field. This study has practical implications as understanding these concerns can help developers and healthcare managers identify potential issues in the planning, developing, and implementing blockchain-based HIE systems. Addressing these barriers would support the widespread use of blockchain-based HIEs in different healthcare settings and facilitate interoperability and connectivity in regional and community health information networks.

## Background

### Blockchain technology

Blockchain is a disruptive technology that could fundamentally change the way companies operate. Blockchain is mainly recognized as the underlying technology for cryptocurrencies, but it is not limited to digital currencies (e.g., Bitcoin, Ethereum, etc.). Blockchain can be used in several industrial sectors such as healthcare, supply chain management, digital rights management, energy, and governments [1]. Blockchain platforms use a decentralized network of distributed nodes to validate transactions and maintain the system's data integrity [2]. The main principles of blockchain are decentralization, peer-to-peer (P2P) transmission, transparency, pseudonymity, irreversibility of transactions, higher security, and computational logic [3]. Thus, a chain of blocks containing operation information avoids having a central repository or middleman to complete transactions. Since there is no central institution, a consensus mechanism is needed by nodes to maintain the consistency of data and preclude systems from failing due to malicious data [4]. Blockchain can have different control mechanisms, such as permissioned and permissionless [5]. In the permissionless blockchain, anyone can join the network as a user and/or node, and the data is publicly available using a public ledger. However, only invited parties can join the network as a user and/or node in the permissioned blockchain. Based on this architecture, some nodes can receive transactions and store data, and some users can read, write, and enter data into the network.

### Blockchain applications in the healthcare

Blockchain is recognized as a potential technology to manage information sharing between healthcare organizations and providers. Blockchain has been suggested as an attractive solution for improving interoperability in healthcare systems, which are generally fragmented [6]. Several studies propose using blockchain-based platforms to securely manage medical information in the healthcare sector [7]. A study examines the impact of blockchain on the healthcare and biomedical industry for security and privacy purpose [8]. Another study proposes a blockchain-based health information exchange (HIE) system for patient monitoring [9]. Blockchain is reported to exhibit organizational benefits (e.g., pharmaceutical supply chain and clinical trials) and patient-related benefits (e.g., personalized healthcare and tracking health data) in healthcare [10]. Using smart contracts is also suggested to enhance the transparency of the entire medical environment, manage access control, and integrate data based on defined patient-provider relationships and data privacy policies [11]. These permissioned platforms can provide a comprehensive log of medical records available to patients and doctors. Each entity can enter, view, track, and audit information stored in a block based on preset role-based access. Federated blockchains are a good fit for the healthcare context where more than one entity is in charge of the system, and a few organizations can control and verify transactions [12].

### Blockchain adoption in healthcare

While a growing number of industry sectors have started using blockchain technology in their business process, the adoption of blockchain-based platforms is still slow in the healthcare sector. According to previous studies, blockchain features such as decentralization, immutability, transparency, and traceability are proposed to improve healthcare data management (e.g., storage, exchange, and access) [13]. Nevertheless, very few blockchain projects have been officially launched in the healthcare ecosystem, and many suggestions still remain in the research and examination phase [10]. Thus, several healthcare organizations and clinicians are still reluctant to adopt blockchain technology as a foundational innovation. The lack of interest in blockchain implementation in healthcare indicates that critical concerns exist for stakeholders regarding integrating this technology into their healthcare systems.

## Research objective

Physicians are one of the most important users and stakeholders of blockchain projects in healthcare. Little is known about how physicians perceive the barriers and challenges of blockchain technology adoption within a healthcare context. Few studies used a literature review approach to identify and categorize the benefits and threats of blockchain technology in healthcare [10]. However, they mainly investigated factors from the healthcare organizations' perspective. This study takes a qualitative methodology to explore values, barriers, and concerns associated with blockchain applications from physicians' point of view. The results can be a step forward in this research direction by investigating the adoption problems deeply from physicians' perspectives. Furthermore, the findings can provide practical implications to managers and developers by highlighting the key concerns that should be well-addressed to encourage the widespread adoption of blockchain.

## Methods

### Research context

HIE systems can be categorized into three groups federated, centralized and blended. In a Federated HIE, each participating organization can control their medical information and respond to queries when patient information is requested (query-based services) [14]. In federated HIEs, a central data repository does not exist, and the HIE only connects different systems by providing record location and patient matching services. A centralized HIE (as a central hub) collects medical information from various healthcare organizations and stores such information in a centralized place to provide access [15]. A blended HIE can store some healthcare information centrally and access other medical information through query-based services. A blockchain-based HIE solution will likely replace the data interfaces between HIE and different Electronic Health Records (EHRs) or other information systems and change how patients might control their own medical data. At the begging of the interviews, these definitions were clearly explained to the physicians who participated in the study to better specify the research context which is blockchain-based HIE solutions.

### Participants

This study aims to identify and categorize the key concerns and barriers to implementing and using blockchain-based HIE from an essential stakeholder (e.g., physicians) in these information-sharing endeavors. Instead of a separate blockchain application, blockchain HIE solutions can be used through their portals or integration with the physicians' EHRs. As a user of HIE, healthcare providers can provide valuable insights about the back-end technology of blockchain for data exchange. The benefit perceptions and risk beliefs of prospective users may affect their future adoption of blockchain HIE solutions. Healthcare providers' adoption decisions could significantly affect what technologies or IT solutions healthcare organizations may use in their practices. Moreover, potential users of blockchain HIE can definitely highlight possible concerns, challenges, and barriers that may refrain them from supporting and using new blockchain-based solutions implemented and promoted by healthcare organizations. Thus, participants were physicians or healthcare providers with various specialties working in different healthcare settings in the United States.

A series of semi-structured, open-ended interviews were conducted online through Zoom (the video conferencing software) to collect and evaluate their perceptions and concerns about using blockchain for HIE purposes. The interviews were mainly directed by a list of structured questions used to facilitate more in-depth conversations and allow a more profound understanding by the researcher. Two inclusion criteria were used consistent with the study objects. The first one was: participants had experience with an HIE network (e.g., regional HIE). The second one was: participants were familiar with the concept of blockchain technology. Using these criteria could help us collect quality and reliable information from healthcare providers aware of the main two topics covered in this research study.

### Design

Although several studies demonstrate general concerns about blockchain technology in healthcare, little is known about physicians' perceptions and experiences around blockchain space and how it enables HIE [7]. Since this phenomenon has not been examined clearly and influential factors have not been determined in repeated studies, a qualitative methodology is conducted using a grounded theory framework to generate deeper insights [16]. The grounded theory uses systematic inductive methods to build concepts and theory from data [17]. Moreover, the Promoting Action on Research Implementation in the Health Service (PARIHS) framework was considered as a basis for selecting interview questions. The PARIHS framework proposes three factors (i.e., evidence, context, and facilitation) that influence the successful implementation of evidence-based practices [18]. Evidence refers to knowledge-based supports for the effectiveness of an intervention (which is blockchain-based HIE in this study). Context explains the environment or setting in which the intervention is implemented (which is the healthcare context in this study). Facilitation posits the technique or process used to smooth change management, such as changing others' attitudes, perceptions, or behaviors to increase the odds of successful implementation [19].

According to Helfrich, Damschroder, Hagedorn, Daggett, Sahay, Ritchie, Damush, Guihan, Ullrich and Stetler [20], the framework consists of interacting core elements. (1) Evidence refers to codified and non-codified sources of perceived knowledge by stakeholders (which are physicians in this study). (2) Context refers to the quality of the environment or setting where the research is implemented (hospital settings in this study). (3) Facilitation refers to a system to help people change their attitudes, habits, skills, and working procedures (blockchain solutions in this study). This framework proposes that a successful implementation is a function of these three factors and their interrelationships. The justification for using the PARIHS framework is to examine how (1) users (i.e., physicians) perceptions and attitudes, (2) hospital-related factors, and (3) blockchain-based solutions and their interrelationships are important in the successful implementation of blockchain-based HIE solutions.

Thus, interview questions are guided by the grounded theory and PARIHS framework. In this study, questions on evidence are related to physicians' awareness and experience with blockchain technology. Questions on context focus on current issues with HIE efforts and physicians' attitudes about the use of blockchain in healthcare settings. Finally, questions on facilitation cover perceived benefits, risks, and suggestions for the successful rollout of blockchain-based platforms for information sharing in healthcare [21]. As the purpose of this study was to characterize physicians' expectations, attitudes, and experiences to help develop a blockchain-enabled HIE, using the PARIHS framework can be helpful to apply the knowledge into practice. The list of questions is shown in Table 1.

**Table 1:** Qualitative interview questions

Category	Interview questions (baseline)
Evidence	Your current awareness of blockchain concepts and characteristics? Your general knowledge about blockchain-based projects in healthcare? Your prior experience with an HIE network? Your prior participation in a blockchain-based HIE solution?
Context	The key problems with information sharing among healthcare organizations? The main issues with exiting HIE efforts? Your opinions and reflections about using blockchain in healthcare? Your attitudes about integrating blockchain into HIE networks?
Facilitation	The potential benefits of using blockchain in HIE efforts? The concerns and risks associated with using blockchain in HIE efforts? Your suggestions about integrating blockchain in HIE efforts? Your recommendations about the successful rollout of blockchain-enabled HIE in healthcare institutions?

### Setting

The interviewed physicians were recruited from four virtual events in the United States in 2021: two clinical and medical informatics conferences at the national level, an annual healthcare symposium, and a yearly medical association summit. These virtual events were conducted at different times of the year. The researcher used purposive sampling to recruit healthcare providers. According to purposive sampling (as a non-probability sampling method), researchers use their judgment when selecting population members to participate in their studies [22]. Based on the inclusion criteria, an invitation email was sent to 81 physicians who met the defined criteria. The purpose and significance of the study followed by a written consent form were sent to potential samples with an email address registered in these events. Two reminders were also sent to them: the first one after two weeks and the second one after one month from the initial email. Finally, 38 respondents agreed to participate in a one-on-one online interview. Physicians who had accepted to be interviewed could avoid participating in this study at any time voluntarily. Two physicians withdrew their acceptance and did not participate in their scheduled interviews. Therefore, the researcher managed to conduct 36 in-depth interviews with physicians.

### Data collection

Data collection and analysis were conducted using the Consolidated Criteria for Reporting Qualitative Research (COREQ) guidelines proposed by previous studies [23]. This study was reviewed and approved by the Institutional Review Board (I.R.B) of the authors' affiliated university, and the data collection was performed confidentially. Although participants in this study were fairly familiar with blockchain, the researcher neutrally reviewed the concept of blockchain technology at the beginning of the interview process to ensure participants could better realize the purpose of this study. The interviewer referred to the baseline questions that covered the main topics under examination in this study and then asked some follow-up questions for clarity purposes or collecting deeper information. The interviewer did not use any positive or negative connotations associated with blockchain not to lead the answers. Each interview lasted approximately 30 to 45 min, depending on the length of answers and the number of follow-up questions.

### Data analysis

The grounded theory analytical techniques were used to analyze the data since there was no prior theoretical framework or coding schema. The interviews were transcribed by two researchers who researched HIE efforts and blockchain platforms in the United States for more than seven years to perform explorative content analysis. The baseline questions were used to systematically review and code the interviews' transcripts until the entire text was covered. The coding schemes used in this study were open, axial, and selective coding procedures suggested by Corbin and Strauss [24] to identify conceptually similar themes mentioned by the interviewees. Open coding is an analytical process that identifies concepts, properties, and dimensions by clearly defining these codes. Axial coding refers to relating categories to identified open codes based on conceptual similarities. Selective coding is the final stage of data analysis. These selective codes represent theoretical constructs formed by connecting the axial codes to provide a theoretical explanation of concerns and barriers to blockchain-based HIE.

Two coders independently coded 38 interviews. During the three phases of coding, the two researchers coded 38 interviews. Using NVIVO-12, the coders proceeded line-by-line, sentence-by-sentence, paragraph-by-paragraph, page-by-page, and section-by-section. This approach ensured that the analysis results were "grounded" in the data [24]. To identify open codes, which we subsequently grouped into categories representing axial codes, we obtained Inter-Rater-Reliability (IRR) of 70% and Cohen’s Kappa of 0.69. Because the IRR metric was less than 75% and Cohen’s Kappa metric was less than 0.75 [25], we repeated the coding process until the agreement metrics fell within the acceptable range. Before repeating the coding process, the coders met to resolve coding disagreements and reach a coding consensus. At the end of this phase, the coders obtained an IRR close to 93% and Cohens Kappa of 0.87, demonstrating that the coding process was reliable and valid across the two coders.

## Results

### Interviewee's characteristics

Table 2 shows the participants' characteristics. IBM SPSS version 27 was used to perform the descriptive statistics. The demographic data highlights that 63% of physicians who agreed to participate in this study were male, and 37% were female. Age range and length of practice (years) were normally scattered, with age range between 45 and 54 years (48%) and length of career between 11 and 15 years (42%) were higher ranges among provided categories. Physicians who participated in this study were from various areas of work and specialty, with family medicine (19%) slightly represented more than others. 31% of respondents mainly practiced in a public hospital, and the majority of the respondents (74%) indicated they worked in an urban setting. Consistent with the inclusion criteria, all 38 interviewees indicated that the hospital/clinic they worked in either implemented or participated in an HIE program (for instance, regional or state-based HIE networks). In addition, all respondents stated that they had participated in an HIE network to share patients' medical records. Thus, the interviewees were familiar with existing HIE projects and their concerns and challenges.

**Table 2:** Sample characteristics

Variable	Categories	n (%)
Gender	Male	24 (63)
	Female	14 (37)
Age	Under 35	4 (10)
	35–44	11 (29)
	45–54	18 (48)
	55–64	3 (8)
	65 or older	2 (5)
How long have you been practicing? (years)	1–5	7 (18)
	6–10	8 (21)
	11–15	16 (42)
	16–20	4 (11)
	More than 20	3 (8)
Specialty	Emergency medicine	2 (5)
	Family medicine	7 (19)
	Psychiatry	1 (3)
	Surgery	5 (13)
	Anesthesiology	4 (10)
	Pediatric	3 (8)
	Internal medicine	6 (16)
	Gynecology	4 (10)
	Urology	3 (8)
	General practitioner	3 (8)
Type of hospital/clinic	Public hospital	12 (31)
	Private hospital	9 (24)
	Academic medical center	4 (11)
	Nonprofit hospital	4 (11)
	Clinics	7 (18)
	Rehabilitation	2 (5)
Healthcare setting	Rural	7 (18)
	Urban	28 (74)
	Community	3 (8)
Has the hospital/clinic you work in implemented or participated in an HIE program? (inclusion criteria)	Yes	38 (100)
	No	0
Have you ever participated in an HIE network to share patients' information? (inclusion criteria)	Yes	38 (100)
	No	0

### Evidence

According to the inclusion criteria, all physicians who participated in this study were generally aware of blockchain concepts and technological underpinning. Around 86% of them mentioned that they were extremely or moderately familiar with blockchain platforms and smart contracts. Around 94% of the participants indicated that they were extremely or moderately familiar with blockchain-based projects in healthcare. The top three examples were blockchain applications in HIE, supply chain management, and tracking counterfeit medical products. 100% of them also stated that they had experience with an HIE network. The top three were direct exchange, lookup exchange, and patient-mediated exchange, where 92% of them were at the regional level, and 8% occurred at the state level. Around 23% of respondents had participated in the development and implementation of a blockchain-based HIE. However, only one of the projects was officially launched when the interviews were conducted. 17% expressed that they had participated in published studies suggesting HIE frameworks using blockchain technology. Finally, 60% reported that they were theoretically familiar with blockchain-enabled platforms designed for HIE.

### Context

#### Critical issues with existing HIE systems

Respondents were asked about the main problems and issues with existing HIE mechanisms used for sharing health information. Relying on grounded theory, coding procedures (open, axial, and selective codes are used to find the common concepts, constructs, and themes. Table 3 demonstrates open codes and common concepts.

**Table 3:** Open codes for problems with existing HIE mechanisms used for information sharing

Open codes	Common concept
Mainly focusing on EHRs, downtime issues, EHR-based exchange, not compatible EHRs, lack of standards, reliability of EHR systems, interoperability issues of uncertified EHRs	EHR-focused exchange
Centralization, central ownership, centralized authority, managed by a middleman	Centralized mechanism
lack of patient-provider interactions, lack of support for care coordination, delays in care delivery	Care coordination
Not transparent, fuzzy purposes of sharing, unclear permission process, lack of visibility of sharing process, ambiguous ownership transfer	Transparency of sharing mechanisms
Privacy concerns, security issues, data breach, a single point of failure, technical security measures	Privacy and security
Data are outdated in HIE databases, unavailability of patient data, incomplete or inaccurate patient information is stored in shared records, lack of real-time access to patient data, data inconsistency, duplicate data	Data quality
Trust issues, trust in recipients, trust-based systems, trust in networks	Trust
Data can be altered, medical records can be manipulated, information can be removed by any entities participating in HIE initiatives	Mutability

Table 4 shows the categories of eight constructs. According to the results, respondents believe that relying too much on EHRs [26] as the centerpiece of HIE systems, mainly using centralized platforms in HIE systems, and the inability of existing HIE systems in maintaining care coordination are the top three problems with existing HIEs.

**Table 4:** Listing of constructs, definitions, anecdotal evidence, and count for issues with current HIEs

Axial Codes (Constructs)	Definition	Sample Quotes	Count
EHR-focused exchange	The extent to which existing HIE systems are functional depending on EHR systems	“Many EHRs are used by various healthcare providers. If the EHR of sender or receiver is down, the exchange will be disrupted.”	32
Centralized mechanism	The extent to which existing HIE systems are centralized	“HIE systems are mainly centralized, meaning that an organization has the final decision-making authorities.”	29
Care coordination	The extent to which existing HIE systems may not support care coordination among multiple entities	“Current HIE mechanisms could take some beneficiaries out of the loop, such as patients.”	25
Transparency of exchange platforms	The extent to which existing HIE mechanisms and their policies may not be transparent	“Sometimes, it is not clear why certain personal health information should be shared.”	23
Privacy and security	The extent to which existing HIE may not use technical security measures to protect health information	“Security safeguards of current HIE cannot 100% guarantee the privacy and confidentiality of data.”	21
Data quality	The extent to which existing HIE systems may not maintain the quality of healthcare data	“Lots of medical records in HIE databases are not useful because they are not current data or they may have missing values.”	20
Trusting relationships	The extent to which existing HIE systems rely on shaped trust in networks	“Now, sharing health information through HIE needs a huge amount of trust in recipients.”	16
Mutability of data	The extent to which health data can be altered and manipulated in existing HIE systems	“It is possible that the content of a health record is changed during sharing process with no notification.”	14

Table 5 depicts the key themes that detail the main issues with existing HIE systems. The core themes are problems with the quality and characteristics of data stored and shared through existing HIEs, issues with central exchange platforms, and concerns about networks of relationships among stakeholders involved in HIEs.

**Table 5:** Selective codes representing problems with existing HIE systems

Selective codes (themes)	Constructs involved	Definition
Data-related issues	Data quality + mutability of data	The extent to which existing HIE systems may not support data quality and maintain immutability of medical records during sharing processes
Exchange platform-related issues	EHR-focused exchange + centralized mechanism + privacy and security + transparency of exchange platforms	The extent to which centralized platforms used in existing HIE systems may not ensure transparency of information-sharing efforts and safeguard the privacy of health information
Network-related issues	Care coordination + trusting relationships	The extent to which existing HIE systems may not help care coordination and increase trusting relationships between involved entities

#### Opinions and attitudes toward using blockchain in healthcare projects

36% of physicians who participated in this study were very positive about the idea of utilizing blockchain in healthcare. With regard to critical issues with existing HIE systems, these respondents believe that “blockchain technology has the potential to solve most of the information-sharing problems in the healthcare space”. 47% were somehow positive about integrating blockchain into HIE networks. The key point repeatedly raised by this group of respondents is that although blockchain could be a good alternative, healthcare organizations should conduct a thorough needs assessment and feasibility analysis before selecting blockchain as a solution. They mainly mentioned that “blockchain is not always the answer to a workflow problem in healthcare” unless strong feasibility analysis shows that functional capabilities of blockchain can fulfill the need (such as ineffective procedures used for information sharing). The remaining 17% held natural opinions about sharing health data using blockchain. The main argument pointed out by this group is that the benefits of blockchain applications in healthcare may outweigh the risks. However, many steps should be managed well in the blockchain system development lifecycle, such as planning and analysis, design, implementation, and support and evaluation phases.

### Facilitation

#### Perceived benefits

Respondents were asked to describe their opinions about the key benefits of using blockchain in HIEs. Table 6 demonstrates open codes and common concepts related to the possible contributions of blockchain to HIE projects.

**Table 6:** Open codes for perceived benefits of using blockchain in HIEs

Open codes	Common concept
ID control, access control, data protection and privacy, security, encryption, cryptographic keys, secure network infrastructure, the anonymity of all users in the blockchain system, blockchain protects user privacy, unhackable networks, identity management, confidential and private transactions, each transaction needs patient’s authorization, user information does not link with personal information, mitigating security and privacy issues	Encryption and control
Data integrity, data integration, helping with data storage and data aggregation, data consistency through immutability	Data management
Better coordination, improved coordination, resolving interoperability conflicts, real-time monitoring of patients’ health status	Interoperable mechanism
Decentralization, empowerment of end-users, accessibility, the democratization of data, new roles defined, more participants, sharing authority, decentralized decision-making power, availability of data in P2P networks	Decentralized system
Digital facilitation of negotiation, automated verification, computerized execution, and enforcement of contracts, automatic identification and authentication of all participants, sharing information through smart contracts without a third-party intermediary	Smart contracts
Transparency of sharing, data provenance, no repudiation, transparent transaction logs, clear mechanisms, no shady activities, transparent data sharing protocols	Transparent protocols
Reduced downtime, the system is always available, optimal uptime, systems less likely will fail to perform, network downtime, no inactive time, not offline period, No system breaks	System uptime
Reduced costs of transactions, no need to pay a middleman, lower transaction costs, lower costs of data preparation, increased speed of transactions, faster data exchange	Speed and cost of transactions

Table 7 shows eight categories of constructs. The top three constructs are encryption and control, data management, interoperable mechanisms. The findings imply that a more robust encryption mechanism and access control are the most important benefit of blockchain, as 92% of respondents stated this in their interviews. The next construct mentioned by 76% of participants is data management and the effect of blockchain on data immutability, data storage, data aggregation, and data consistency. The third construct stated by 71% of respondents is the interoperable structure where blockchain could enhance collaboration among various entities in the healthcare industry.

**Table 7:** Listing of constructs, definitions, anecdotal evidence, and count for perceived benefits of blockchain-based HIEs

Axial Codes (Constructs)	Definition	Sample Quotes	Count
Encryption and control	The extent to which blockchain is secure to encrypt data, control access, and protect the privacy of health information during transactions	“Blockchain functions based on a secure network infrastructure that ensures confidentiality and privacy of sensitive health information.”	35
Data management	The extent to which blockchain can improve data storage, data aggregation, and data consistency	“Systems using blockchain can integrate data from different sources (such as patients) and effectively distribute them across different entities.”	29
Interoperable mechanism	The extent to which blockchain enables to communicate and exchange usable data across different parties in a network	“Blockchain technology can overcome the interoperability barriers of the current HIE systems and facilitate interoperable communications between different healthcare organizations.”	27
Decentralized system	The extent to which blockchain can distribute authority and decision-making power across various entities in a network	“Relying on a P2P network, more roles are defined, and more people will be in the loop of information provision, verification, and exchange.”	23
Smart contracts	The extent to which blockchain enables smart contracts to digitalize, control, and automate transactions	“Blockchain-powered networks can run programs and codes to verify and execute transactions automatically when predetermined conditions are met.”	20
Transparent protocols	The extent to which blockchain uses protocols to increase the transparency of information sharing transactions	“Users can visibly track where health data comes from, what happens to it, where it goes over time, and why it is shared.”	17
System uptime	The extent to which blockchain platforms could be free from network downtime and inactive time	“In the last decade, only blockchain platforms proved the claim of enhanced system uptime.”	15
Speed and cost of transactions	The extent to which blockchain could increase the effectiveness and efficiencies of information-sharing efforts	“blockchain is seen as being better than previous technologies in terms of reducing costs and improving the speed of transactions.”	11

Table 8 depicts the key themes that represent the main benefits of blockchain-based HIEs. The core themes are benefits resulting from innovative technological features, collaborative ecosystem, and system performance. The theme of innovative technological features includes using stronger encryption and control, smart contracts, transparent protocols, and data management. Collaborative ecosystem reflects how blockchain can enhance communication and coordination through the interoperable mechanism and decentralized system. The System performance theme describes how blockchain can increase the effectiveness and efficiency of transactions.

**Table 8:** Selective codes representing benefits of blockchain-based HIEs

Selective codes (themes)	Constructs involved	Definition
Innovative technological features	Encryption and control + smart contracts + transparent protocols + data management	The extent to which blockchain uses innovative features to improve encryption, access control, data management, transparency of sharing procedures, and automatic verification and execution of transactions
Collaborative ecosystem	Interoperable mechanism + decentralized system	The extent to which blockchain uses decentralized platforms to improve interoperability standards in healthcare
System performance	System uptime + speed and cost of transactions	The extent to which reliable blockchain-based networks could increase the effectiveness and efficiency of information sharing transactions

#### Perceived concerns

Then, respondents were asked to evaluate possible concerns and risks associated with using blockchain in HIE. Table 9 demonstrates open codes and common concepts regarding problems of blockchain-based HIEs.

**Table 9:** Open codes for perceived concerns associated with using blockchain in HIEs

Open codes	Common concept
Familiarity, knowledge, exposure, understanding, insights about the system	Awareness
Outsourcing, outsourced platforms, in-house, third-party supplier, system design phase, contracting with a vendor, designer contract, contract with an outside provider	Selection decision
Regulatory issues, unclear rules and regulations, lack of regulatory guidelines, lack of supporting laws, not regulated area, not aligned with HIPAA regulation privacy compliance metrics	Regulations and laws
Not doable projects, not feasible efforts, lack of support from managers, not aligned with hospital strategic directions, not backed by healthcare organizations' policymakers	Feasibility
Involvement of various entities, collaboration issues, patient roles, needs for a strong network of relationships, communications issues among different beneficiaries	Stakeholders participation
Blockchain architectures, public or private models, authority agency, various platforms, model conflicts, right model in healthcare, different model structures, different verification procedures, consensus algorithm	Blockchain model types
Resource allocations, workflow changes, installing a new system, integrating blockchain into healthcare, system installation, network infrastructure, implementation duration, implementation cost	Implementation
Lack of adoption in the market, competitors' adoption, other providers' acceptance, not popular in the market yet, challenge with the widespread adoption of blockchain, state of blockchain adoption	Industry traction
Lack of technical trust, untrustworthy technical underpinning, technological immaturity, unreliable foundation, technology in its infancy, instability, unscalable, errors	Trust in technology
Not easy to use, complicated foundations, confusing protocols, difficult platforms	Complexity

Table 10 shows ten categories of concerns. The top three constructs are lack of knowledge about blockchain technology, implementation issues, and regulatory issues. 89% of respondents mentioned that the most important concern is lack of knowledge, as the majority of users in healthcare are still not familiar with blockchain technology and its applications. 84% of respondents believed that implementation issues could be an essential barrier to blockchain-based HIEs. 78% of interviewees highlighted that a lack of supportive rules and regulations to deploy blockchain in healthcare would discourage users from integrating this innovative technology into their routine practices.

**Table 10:** Listing of constructs, definitions, anecdotal evidence, and count for perceived concerns of blockchain-based HIEs

Axial Codes (Constructs)	Definition	Sample Quotes	Count
Lack of knowledge about blockchain technology	The extent to which healthcare providers may not be aware of blockchain applications in healthcare and why they could be useful	“Most of my colleagues don’t know what blockchain is… and how it can be used in healthcare practices.”	34
Implementation issues	The extent to which healthcare organizations may not have required resources and effective planning to integrate blockchain into routine healthcare practices	“Some managers are terrified by technical issues, time, cost, training, and value-added of blockchain projects.”	32
Regulatory issues	The extent to which physicians believe that using blockchain-based HIE is not fully regulated	“I don’t think blockchain applications are completely regulated in the healthcare space and still need laws to support it.”	30
Selection decision	The extent to which selecting blockchain platforms in healthcare is challenging when there are various alternatives (i.e., outsourced, in-house)	“Many outside providers can design blockchain-based exchange methods, but some managers believe they need to develop blockchain platforms in-house.”	27
Feasibility issues	The extent to which blockchain projects may not be a feasible effort from organizational perspectives	“I think blockchain integration is not a feasible endeavor for healthcare managers; that’s why many of them do not support these kinds of projects.”	25
Lack of collaboration among various stakeholders	The extent to which various stakeholders may not be willing to collaborate in blockchain-based HIE	“A number of entities got to collaborate in blockchain HIE, such as patients…. I am not sure if all agree on the role of patients in transactions.”	23
Blockchain model types	The extent to which healthcare providers may not deploy the right blockchain models (among various architectures) which is suitable in the healthcare domain (e.g., public, private, hybrid, federated)	“There are some blockchain architectures, but it is challenging to realize which one could be the most secure model with a robust infrastructure for information exchange in healthcare.”	21
Complicated system	The extent to which using blockchain may not be free of effort for users	“Technological foundations of the blockchain and smart contracts do not seem to be easy.”	18
Network effects	The extent to which competitors (such as large healthcare organizations) may not adopt blockchain	“We are scared that we are the only one [27] adopted blockchain… as a matter of fact, competitors' responses are not clear.”	15
Lack of trust in blockchain technology	The extent to which healthcare providers believe that blockchain may not be a trustworthy, reliable, and error-free technology	“I think blockchain is still a new technology, still unstable and not scalable.”	12

Table 11 depicts the key themes that represent the possible concerns of blockchain-based HIEs. The core themes are individual-related issues, technology-related issues, organizational-related issues, and market-related issues. This finding describes that concerns and risks associated with blockchain-based HIEs can be investigated from four perspectives: (1) how does an individual user think of blockchain? (2) what technological factors may impede adoption? (3) what organizational factors can be barriers to the use of blockchain? (4) what external factors may hinder the successful rollout of blockchain?

**Table 11:** Selective codes representing concerns about blockchain-based HIE

Selective codes (themes)	Constructs involved	Definition
Individual-related issues	Lack of knowledge about blockchain technology + lack of trust in blockchain technology	The extent to which physicians may not be familiar and aware of blockchain platforms and may not trust in the reliability of this technology
Technology-related issues	Complicated system + Blockchain model types	The extent to which developing the right type of blockchain architecture compatible with healthcare would be challenging, and that blockchain model may not be easy to use for HIE purposes
Organizational-related issues	Feasibility issues + selection decision + implementation issues	The extent to which healthcare organizations believe that blockchain projects may not be feasible, selecting blockchain-platform is challenging, and the required resources may not be available for a successful rollout
Market-related issues	Network effects + lack of collaboration among various stakeholders + regulatory issues	The extent to which regulations may not support blockchain applications, the healthcare market may not be receptive, and stakeholders may not fully participate in blockchain projects

## Discussion

### Principal findings

#### Perceived concerns and risks with conventional HIEs

The respondents expressed various concerns related to current HIE systems, such as direct emails or lookup models. The first category of problem is related to data quality in HIE databases. Storing incomplete, duplicate, inaccurate patient information in shared records and lack of real-time access to patient data could have detrimental consequences such as wrong treatments and incorrect medicine. Moreover, medical records can be altered, manipulated, or even removed by any entities participating in HIE initiatives. This issue poses an essential risk to the reliability of HIE databases. The second theme has been shaped around skepticism about the conventional exchange platforms. The existing HIEs are mainly conducted through EHRs, which are centralized platforms, and data exchange occurs in a central ownership environment. One common issue with EHR-based exchanges is the lack of standards because healthcare providers may use different uncertified EHRs. The other possible issue is ambiguous data ownership and the lack of visibility of sharing process, which arises due to centralization. In line with the literature, centralization also may increase the odds of privacy invasion, security issues, and data breaches [28]. Since a central repository is more likely to encounter a single point of failure, technical security measures may not ensure health information privacy. The last theme is related to care coordination and trusting relationships in HIE networks. Consistent with previous studies, the central HIE systems work well when various central EHR systems collaborate through a trusting environment [29]. For instance, if a provider uses his/her direct email to share patient information with an unaffiliated counterpart, first, the recipient should be known and trusted. The trust process does not determine by technology, and it is a human factor. The sender should know who the recipient is and whether he/she is trustable. Thus, a lack of trust in an HIE network may prevent entries from collaboration and result in delays in care delivery.

#### Perceived benefits of blockchain

Respondents pointed out several benefits expected from blockchain applications in HIEs. These benefits could mainly address the key challenges and risks with conventional HIE systems. First, the benefits stem from innovative technological features of blockchain as a radical change that could substitute the business model of established organizations. Blockchain technology offers unique characteristics, such as dual-key encryption that adds a robust security layer to conventional HIE systems. Consistent with previous studies, the decentralized network of distributed nodes can add more security and visibility to transactions because it doesn’t have data breach risks possible with centralized EHR systems [30]. Since a paramount concern associated with HIEs is privacy and security issues, implementing a system that can minimize data collection issues, unauthorized access, and secondary use of data would be a competitive advantage for healthcare organizations. Moreover, a decentralized computing platform processes complex smart contracts to serve a myriad of use cases. Smart contracts can digitally define the terms of the agreement between information seekers and information providers that can be performed automatically without a third party. Blockchain-based HIE can utilize smart contracts to integrate data based on the creation of patient-provider relationships. Thus, blockchain can add transparency into the data sharing process as all entities (including patients) can view what health information is collected and shared, why it is exchanged, and to whom it is transferred? As highlighted by previous studies, smart contracts contain contracts and requirements for data ownership, access control, and authorization [31]. Smart contracts can automatically enforce transactions to obtain consensus from providers and patients before giving viewership to a third party. Smart contracts can reduce transaction and legal, operational, and infrastructure costs in the absence of a middleman. Furthermore, smart contracts can replace trust with automatically-executed terms and conditions according to personal data privacy policies, health data record policies, and third-party involvement policies.

Second, the decentralized nature of blockchain can also improve interoperability standards in healthcare. Decentralization based on distributed nodes can enhance the empowerment of end-users and contribute to the democratization of data. Blockchain-based HIE can enable various nodes to communicate with each other. Patients and providers can have their own nodes to participate in the P2P network. Each node has some responsibilities, such as reading, writing, and entering content. Caregivers can review the historical interaction between healthcare professionals and patients in real-time. Blockchain-powered HIE could provide a comprehensive log of medical records available to patients and enables them to accept or reject relationships with healthcare providers. Consistent with previous studies, blockchain platforms can resolve interoperability conflicts between involved entities through real-time monitoring of patients’ health status and enhanced care coordination [32].

Third, previous studies propose that blockchain could improve system performance by increasing the effectiveness and efficiency of information-sharing transactions [33]. Historically, blockchain networks have proved that they could significantly reduce system downtime. Centralized HIEs could suffer from network downtime and inactive time. However, one premise of blockchain is high uptime with fewer to no system breaks that could provide high availability [34]. The distributed ledger enables each node to keep a copy of the entire ledger and administer the chain independently. Thus, if one node fails, no disruption occurs, the remaining nodes continue operating well. Blockchain-based HIEs could reduce the legal and operational costs of exchange transactions because there is no need to pay an intermediary. These platforms also increase the speed of transactions and provide faster data exchange since smart contracts make medical records accessible in real-time. For example, when a healthcare provider adds new health information or a patient shares a portion of medical records with an authorized third party, automated information is made available in the network. The blockchain HIE could instantaneously allow a patient to recover medical records from the provider node or authorize a third party to access their shared data on demand.

#### Perceived concerns and risks with blockchain

Respondents also highlighted some concerns and risks associated with the use of blockchain in HIEs. The first source of concerns is related to individual perceptions about blockchain technology as a whole. Since blockchain is still new, many people (including healthcare professionals) have little knowledge about platforms designed based on this radical technology. Lack of knowledge may also lead to a lack of trust because many people believe that blockchain is in its infancy and is not ready to be used in a sensitive environment such as healthcare [35]. Physicians are professionals in a specific body of knowledge, but they are not necessarily technical experts. Insights and understandings about advanced technology are a function of exposure to technological innovations. However, very few hospitals have already integrated blockchain into their practices; thus, many physicians are still not wide-open to blockchain applications. Respondents expressed that lots of physicians might also confuse blockchain applications in healthcare with cryptocurrency (such as bitcoin blockchain). Since most people only hear negative news when it makes it to the mainstream media and several media coverage highlights illegal use of bitcoin in the dark market, it may affect the general perceptions of physicians about blockchain technology.

The second type of concern is related to the technical aspect of blockchain. Physicians aware of blockchain applications in healthcare may believe that this technology is not easy to use. Participants mentioned that the complexity of blockchain technology (such as mining, encryption protocols, and nodes) is a considerable barrier to adoption. Physicians might believe that using blockchain to exchange health information is complicated, and they may not be able to handle all technical requirements to control their assigned nodes. The other technical concern is that developing the right blockchain architecture compatible with the healthcare domain would be challenging. Different model structures use different verification procedures and consensus algorithm. For instance, public or permissioned blockchain uses different ways of controlling and showing transactions [36]. Permissionless maybe is not a good option for blockchain HIEs since anyone can read and write on the network, and all transactions (i.e., exchange records) are made public with individual anonymity. Permissioned blockchain can be based on different architectures, such as private and federated. In the private blockchain, verification is done by just the owner of the blockchain. That single “highly trusted” organization can control the rights of reading and writing data on the blockchain. However, it would be challenging to align many organizations to use the same blockchain. As proposed by other studies, in the federated (or consortium) blockchain, more than one organization is in charge of the system [37]. Thus, it can be a better fit for HIE initiatives where multiple entities control separate nodes to share information between different organizations. Complying with the technical aspects of various blockchain architectures could be another source of concern for healthcare professionals.

The third category of concern is related to organizational strategies and practices to select and implement blockchain technology. Respondents stated that a new system (e.g., blockchain HIEs) may not always solve a data-sharing problem between healthcare organizations. As highlighted by previous studies, many interoperability issues can be rooted in ineffective procedures or partial use of conventional HIEs [38]. Blockchain HIE initiatives may be terminated because they are not aligned with hospital strategic directions or are not supported by policymakers of healthcare organizations. Hospital leaders may consider quantifiable data and reports (data-driven decisions) or use their subjective judgments to conclude that initial costs and long-term costs associated with maintaining, supporting, and enhancing the blockchain system make blockchain HIEs unfeasible. Another organizational issue in system acquisition is selecting the right application that meets the organization’s needs. In the design phase, managers should finalize the make/buy decision by choosing between designing an in-house blockchain platform or contracting with an external supplier. Conducting a cost–benefit analysis can lead to contracting with an outside blockchain developer, designing a blockchain platform in-house, or purchasing from a blockchain vendor. When a healthcare organization passes the selection phase, they enter the next important phase, the implementation stage [26]. Implementation is characterized by allocating the required resources, changing conventional data sharing workflow, installing a new blockchain system, testing the blockchain-HIE platform, integrating blockchain into healthcare practices, developing network infrastructure, planning implementation duration, and calculating implementation costs (i.e., recurring and non-recurring costs). Arranging these initiatives needs strategic planning, human inputs, infrastructure resources, and support from management.

The last class of concern refers to market-related issues. The success of blockchain-powered HIEs is also dependent on external factors that may not be under the direct control of a healthcare organization. One dimension of the market-oriented concerns is how the use of blockchain in healthcare is regulated. Respondents generally expressed that rules and regulations governing blockchain applications in healthcare are not very clear. Consistent with previous studies, since laws and regulations usually lag behind technological advancements, a lack of regulatory guidelines and supporting laws would be a source of concerns [10]. Respondents were not sure that blockchain-based HIEs were aligned with the Health Insurance Portability and Accountability Act (HIPAA) privacy and security compliance metrics. The other external risk is the industry traction and lack of blockchain adoption in the market. HIE initiatives should occur in a network of providers, and it would be impractical if only a few healthcare organizations joined the network. Thus, the value of network membership to one user is positively affected when other users join and enlarge the network. The value in this context can be effective data-sharing across several providers, resulting in better treatment, care planning, and patient safety. Consistent with Alabi [39], such markets refer to exhibit network effects or network externalities. Besides network effects, lack of collaboration among various stakeholders (e.g., patients, providers, insurance companies, etc.) can cause risks. It would be possible for many stakeholders to join the network, but few of them may fully participate in blockchain projects through their nodes. Lack of stakeholders' participation can lead to delays in data exchange in the network. In particular, the role and responsibilities of patient nodes in transactions are still not clear for other beneficiaries. For instance, if a provider adds new medical exam records to the blockchain but the patient node does not accept/reject relationships to verify the transaction, it will slow down the work process, and the data may not be recorded in the ledger.

#### A guiding framework

**Fig. 1:**
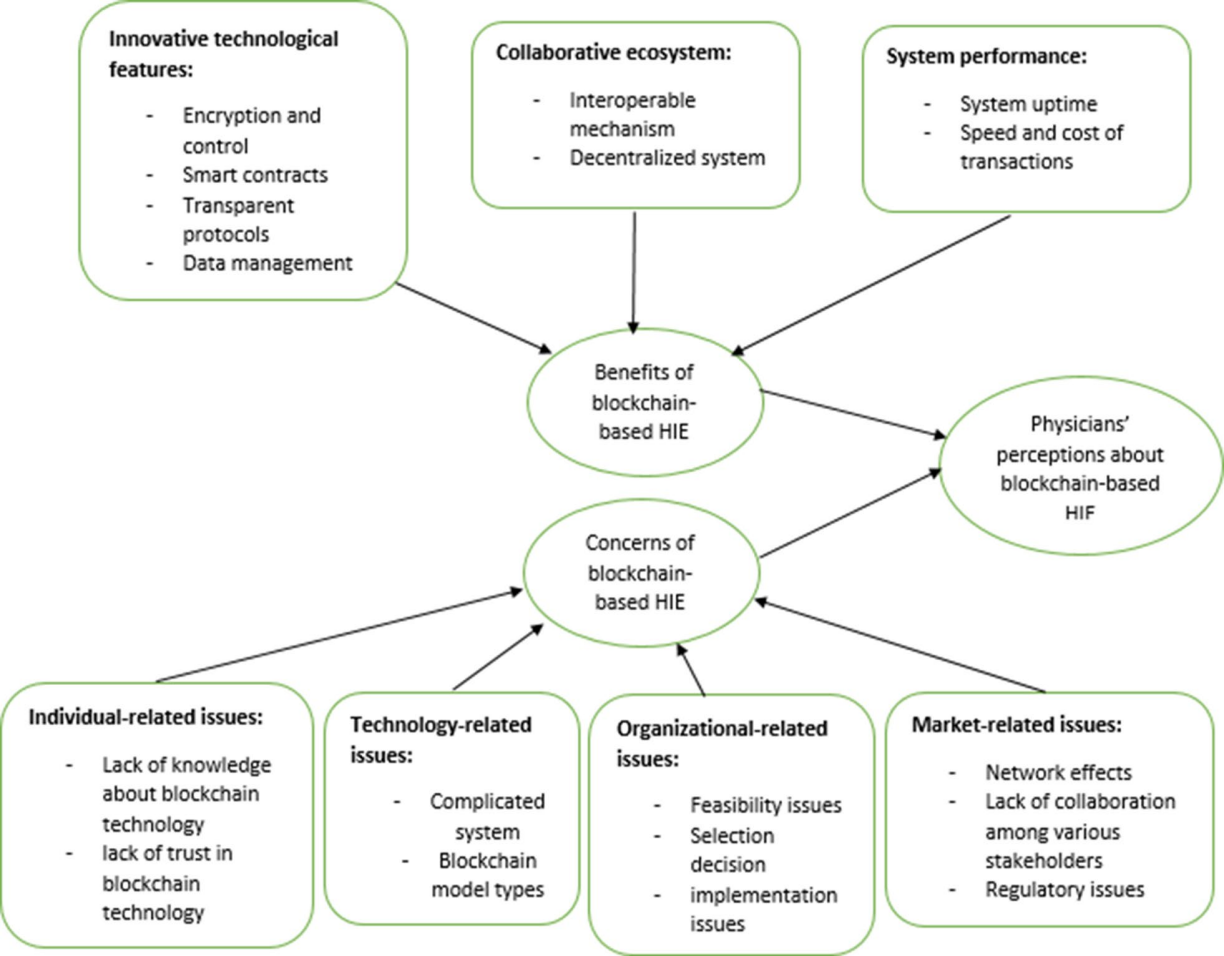
Guiding framework

The findings imply that physicians expect several benefits from blockchain HIE; however, concerns and risks are also not negligible. The calculus of perceived benefits and risks may determine value from blockchain applications in information exchange among healthcare providers. Thus, if blockchain delivers less added value than conventional HIE methods, healthcare professionals will negatively favor blockchain-based HIE. This study contributes to the current discussion on blockchain in healthcare by providing a better picture of what benefits and concerns may shape physicians' perceptions about blockchain-based HIE initiatives. Figure 1 displays a guiding framework to show the main themes, categories, and concepts resulting from interviews.

#### Managerial contributions

In this section, practical implications of the results are discussed. These practical implications can be helpful for both healthcare managers and blockchain system developers. The first managerial contribution is that the pre-adoption phase is as important as the implementation stage for leveraging successful blockchain solutions. Pre-adoption is a critical step because lack of awareness and familiarity of users (physicians in particular) with the concepts and bases of blockchain is raised as an essential concern. In the pre-adoption phase, healthcare organizations need to create awareness, evaluate the possible disruptive effect of blockchain technology on current workflow and practices, and conduct preliminary exploration [40]. Thus, managers may begin with the fundamentals (like learning blockchain) and familiarize physicians with basic blockchain terms and technical specifications. Providing a glossary of the most popular and essential jargon, terms, and shorthands can result in higher education and blockchain guides. Since healthcare professionals generally lack insight into the world of blockchain solutions, exposure through seminars, workshops, or online hands-on training could allow potential users to better understand the business value of blockchain-enabled HIEs.

The second recommendation is that healthcare managers revisit their organizational strategy and address what they currently do regarding information exchange, why blockchain is required, what blockchain architecture is the best option, and how they can implement it. The unauthorized sharing of sensitive health information can erode public trust in healthcare systems [41]. Healthcare organizations can encourage increased adoption of blockchain for secure and interoperable HIE. Blockchain system developers can play an essential role in designing blockchain-based HIE that improves interoperability. Blockchain system developers need to develop federated blockchain models for HIE that enable healthcare organizations to cooperate with various entities. Then, Blockchain system developers can define technical standards and collectively urge regulators to articulate compliance requirements to encourage scaled blockchain solutions for HIE [42]. Handling regulatory uncertainty such as compliance with HIPAA to minimize data breach risk could significantly support organizations in adopting blockchain in the healthcare industry. When several entities participate in a federated blockchain for information sharing, it allows healthcare strategists to develop legal frameworks for different use cases and impact the development of the regulation in their favor.

The third practical contribution is how healthcare organizations can make sense of the potential value of blockchain-enabled HIEs versus conventional HIE models. Mangers and strategies can communicate the problems and risks attached to conventional HIEs to physicians. Then, the key advantages of using blockchain-based HIEs should be highlighted in organizations’ communication channels (such as an intranet or forums) to clarify the value-added of blockchain applications. Finally, they need to express possible blockchain-related risks and how they can address them. For instance, physicians may be worried about the additional workload they may encounter when checking the medical history in a severe condition or strict regulations that make it difficult to reap blockchain’s potential and, then, to obtain the promised value of blockchain [31]. Clear organizational policies and guidelines should be in place to provide information about how managers figure out the compliance, setup, integration, education, workload, and live support.

Finally, dependency on intra- and inter-organizational collaborations is a significant barrier to the successful implementation of permissioned blockchain [43]. Any organizational chain will entail a certain amount of risk. Integrating blockchain is a radical change that fundamentally transforms business models and may involve higher risk for healthcare managers and users. Healthcare organizations that retrofit their strategies with blockchain-based HIEs may strongly believe in decentralized technology. They may want to be a technology leader by developing technological standards and providing a network for other organizations unfamiliar with blockchain to join. They may consider blockchain as a technology to redesign the organizations’ current business model to remain competitive in a fierce market. Cost reductions and efficiency gains provided by blockchain can provide the opportunity to improve healthcare organizations’ market positioning. Thus, other affiliated or unaffiliated organizations may be willing to collaborate within a network to be involved in information-sharing activities. This dynamism can create an environment enabling all healthcare organizations in the blockchain network to obtain the value-added of the technology. Moreover, innovative healthcare organizations can form a blockchain unit (including passionate physicians and IT department representatives) in which they work on the adoption of blockchain, learning competencies, and sharing with other physicians under strong management support.

### Study strengths

This qualitative study was mainly conducted to discover physicians’ perceptions, concerns, and expectations about using blockchain in HIE systems. Thirty-eight physicians who were familiar with HIE and blockchain technology were selected to provide valid and relevant insights. The study design has some strengths. First, the interviewees were sampled from various specialties, experience levels, workplaces, healthcare settings, with diverse personal demographics, such as age and gender. This approach could increase data richness by identifying themes generated from interviewing physicians with different backgrounds. Second, the participants of this study were reasonably familiar with HIE and blockchain principles, which was not likely seen in previous studies. Third, this research is among the first qualitative studies to translate physicians’ experiences and perceptions to specific suggestions for the successful rollout of blockchain-based HIE in healthcare organizations.

### Limitations and future studies

This study has several limitations. First, the findings of this study, which focused on a group of physicians in the United States, may not be generalizable to other countries. Moreover, 38 participants cannot entirely represent the opinions and perspectives of the entire physician population. It would be interesting for future studies to examine the perceptions of more physicians in other countries with different technology infrastructures. Second, the participants in this study were selected using purposive sampling based on particular inclusion criteria to obtain meaningful and practical insights. Thus, respondents were selected on the basis of their familiarity with HIE efforts and blockchain applications in healthcare. Thus, future research can extend this study using other sampling methods (e.g., probability sampling) to include a more diverse sample of physicians. Third, this study attempted to explore the opinions and attitudes of physicians, not other HIE stakeholders (such as health authorities). It can be of interest for future research to investigate the perceptions of other stakeholders, such as patients or health care policymakers. Fourth, the coding procedure consisted of two researchers using NVIVO software to compare and contrast the interview transcripts, identify themes, and choose exemplary quotes. Other researchers may have identified different concepts, themes, or quotes. Future researchers can extend this study and mitigate researcher bias by regular discussions with a larger research team. Finally, this qualitative study identified several constructs (perceived benefits and risks) that may affect physicians’ intentions to use blockchain-based HIE in their practices. Future quantitative research is needed to examine the significance and importance of these constructs in the successful implementation of blockchain at hospital-based levels. Moreover, further studies are required to empirically examine the relationships between variables exhibited in the guiding model to measure the prediction power of the proposed model.

## Conclusions

Recent studies suggest that blockchain has the potential to provide robust and safe infrastructure for healthcare projects, such as HIE efforts. The main objective of blockchain-powered HIE is to provide a way to store and share health information more effectively, securely, and efficiently. Blockchain technology also raises concerns and skepticism that impede it from finding appropriate paradigms for use in HIE networks. This study is an attempt to deeply explore, identify, and categorize physicians' perceptions about the use of blockchain-based HIE. This qualitative study is among the first steps to address the blockchain impact on the healthcare industry for information-sharing purposes. The results indicate that although blockchain technology can yield several benefits (e.g., innovative technological features, collaborative ecosystem, and system performance), its applications in healthcare are still in their early stages. The strong beliefs about the individual, organizational, technological, and market-related issues covered in the analysis show that healthcare blockchain applications continue to be an emerging field. The results can be leveraged to provide insights into physicians’ opinions about blockchain's practical advantages and common concerns underlying the anti-blockchain arguments. Consistent with the identified themes, several solutions are suggested to help shape strategies for blockchain rollout in HIE networks. To include large-scale blockchain platforms in healthcare vision, healthcare managers and policymakers should exert significant effort to highlight potential benefits and address possible risks by devising supportive strategies. Providing effective education campaigns to the physicians' community could be a useful means to increase their awareness of blockchain technology, the most appropriate applications, and its possible effects. This study can serve as a framework for future empirical research designed to assess blockchain technology applications in the healthcare field.

## Data Availability

The datasets used and/or analyzed during the current study available from the corresponding author on reasonable request.
